# Draft Genome Sequence of the Potassium Feldspar-Solubilizing Bacterium *Ensifer adhaerens* L18

**DOI:** 10.1128/genomeA.00199-17

**Published:** 2017-04-27

**Authors:** Qingjing Peng, Langbo Yi, Qingzhong Peng, Yuyuan Peng

**Affiliations:** a College of Chemistry and Chemical Engineering, Jishou University, Hunan Jishou, China; b College of Biology and Environmental Sciences, Jishou University, Hunan Jishou, China

## Abstract

*Ensifer adhaerens* L18, isolated from potassium feldspar mining area soil, was found to be capable of solubilizing K from an insoluble K-bearing mineral source. Here, we report the draft genome sequence and annotation of the feldspar-solubilizing bacterium *Ensifer adhaerens* L18. These data provide the basis to investigate the relative impact of bacteria in feldspar solubilizing and the molecular mechanism of the potassium feldspar’s dissolution.

## GENOME ANNOUNCEMENT

Potassium (K) is an important macronutrient for plant growth and development, and K is a limited element due to its lesser availability in soluble form in the soil (1, 2). However, the most common mineral components of K, at 90 to 98%, are low-grade K feldspar and mica (3). Currently, chemical processes for making use of K feldspar are not eco-friendly and are inefficient. In order to overcome these problems and meet the potassium fertilizer demands, bioleaching can be developed by capitalizing the low-grade potassium minerals (4). Our previous studies showed that *Ensifer adhaerens* L18 could effectively extract potassium ion from K feldspar under the optimized culture conditions (5).

To gain more insight into the bacterial dissolution mechanisms of K feldspar, a highly pure genomic DNA sample of strain L18 was sequenced using the MiSeq (Illumina) system. A paired-end library with an insert size of 550 bp and a mate-paired library with an insert size of 5,000 bp were constructed for the 454 sequencing, and 1,445 Mb (195-fold coverage of the genome) and 551 Mb (31-fold coverage of the genome) of reads were obtained, respectively. These reads were assembled using Newbler version 2.8 (Roche), the scaffolds were connected according to 5-kb mate-paired relationships, and gaps were filled using the GapCloser with read mapping information. All of the assembled contigs were subjected to gene prediction using the Glimmer 3.0 (6). The metabolic pathways were examined through the Gene Ontology (GO) and the Cluster of Orthologous Groups (COG) database. The tRNAs and rRNAs were identified using tRNAscan-SE and RNAmmer 1.2, respectively (7, 8). Other noncoding RNAs, including microRNA (miRNA), small RNA (sRNA), and small nuclear RNA (snRNA), were analyzed using the Infernal software and the Rfam database (9, 10).

The draft genome sequence of strain L18 was 6,874,129 bp in length, which consisted of 22 contigs and 12 scaffolds, and the genome of strain L18 had a G+C content of 62.8%. From the genome comparative analysis results, the genome consisted of 6,552 genes with an average length of 897.8 bp. The total length of the genes was 5,882,442 bp, which made up 85.6% of the genome. According to tRNAscan-SE and RNAmmer, 51 tRNA genes with a total length of 4,003 bp can be found, which make up 0.058% of the genome. In addition, one rRNA operon and 22 other ncRNAs were also determined in the genome. Additionally, strain L18 carried a number of predicted protein-coding genes: 5,116 CDSs were involved in the 30 functional GO groups, and 5,664 CDSs were involved in the 21 COG groups, among which 377 CDSs encoded inorganic ion transport and metabolism. A detailed comparison of strain L18 genome with the genomes of other *Ensifer adhaerens* strains has shown that strain L18 has 67 specific genes, which may provide some insights into the molecular mechanism about solubilizing K.

### Accession number(s).

The draft genome sequence of *Ensifer adhaerens* L18 has been deposited at DDBJ/EMBL/GenBank under the accession number LAZS00000000. The version described in this paper is version LAZS00000000.
